# Preventing Translational Scientists From Extinction: The Long-Term Impact of a Personalized Training Program in Translational Medicine on the Careers of Translational Scientists

**DOI:** 10.3389/fmed.2018.00298

**Published:** 2018-11-09

**Authors:** Margot M. Weggemans, Marieke van der Schaaf, Manon Kluijtmans, Janet P. Hafler, Norman D. Rosenblum, Berent J. Prakken

**Affiliations:** ^1^Center for Research and Development of Education, University Medical Center Utrecht, Utrecht, Netherlands; ^2^Center for Education, University Medical Center Utrecht, Utrecht, Netherlands; ^3^Center for Academic Teaching, Utrecht University, Utrecht, Netherlands; ^4^Teaching and Learning Center, Yale School of Medicine, Yale University, New Haven, CT, United States; ^5^Eureka Institute for Translational Medicine, Siracusa, Italy; ^6^Department of Pediatrics, Faculty of Medicine, University of Toronto, Toronto, ON, Canada

**Keywords:** translational medicine, clinician-scientist, translational scientist, translational research, training, education, personal professional theory, program evaluation

## Abstract

Far too much biomedical research is wasted and ends in the so called “Valley of Death”: the gap that exists between biomedical research and its clinical application. While the translational process requires collaboration between many disciplines, current translational medicine focuses on single disciplines. Therefore, educational pathways that integrate clinical and research skills in interdisciplinary and interprofessional contexts are needed. The Eureka institute (http://www.eurekainstitute.org/) was founded to address these issues. The institute organizes an annual 1-week international certificate course to educate professionals in the domains of translational medicine.

**Study design:** This study set out to investigate the impact of the Eureka certificate course on the alumni, focusing on their ability to engage in translational activities and thus become more proficient translational professionals. An explanatory, mixed-methods study was executed.

**Data collection:** A questionnaire was distributed to collect quantitative data on the number of alumni who were able to apply what they learned during the Eureka course and engage in translational activities. Questionnaire data were also used to inform the semi-structured interviews that were conducted subsequently.

**Results:** Fifty-one percent of the alumni reported that participating in the Eureka course played a role in their decision to change to a different job or in the way they were accomplishing their everyday work. Ten conditions for change that either hampered or supported the Eureka alumni's engagement in translational research activities were identified. Further, the learning outcomes of the Eureka course that impacted the alumni's professional activities were explored using Personal Professional Theory (PPT). The insight that alumni gained in the full translational spectrum and stakeholders involved stimulated reflection on their own role within that pathway. Further, according to the alumni, the course provided them with the skills and confidence to pursue a career as translational professional. These learning outcomes, in combination with conditions that supported alumni's engagement in translational activities, such as supportive professional partners, opportunities to network or collaborate, and a translational work environment, contributed to the large number of alumni that were able to engage in translational activities.

## Introduction

For several decades, concerns have been raised about the large amount of biomedical research that end in the so-called “Valley of Death”: the gap that exists between biomedical research and its clinical application (1, 2). Modern medicine fails to translate innovations at the bench to tangible products at the bedside, leading to an estimated waste in research of 85% of all research funding (3). Although some waste in research is inevitable, the real concern is that a large part of this waste is due to structural issues in the research ecosystem and could be avoided (3–5). The cause of this problem is multidimensional and crosses the domains of academia, industry, and government (6, 7). Indeed, academic, commercial, and political interests often influence decisions about what is studied and how this research is executed, while users of research evidence, such as patients and clinicians, are rarely involved in these decisions (4).

To better align biomedical research with clinical needs and allow for translation of discoveries to clinical practice, there is a need for improved collaboration between all disciplines that are part of the translational process (4, 6) This requires central figures with a full understanding of the translational spectrum, who are able to integrate the perspectives and needs of all involved. These translational scientists—either PhDs with an interest in clinical research or clinician-scientists—need to have a broad set of knowledge and skills to help breaking down the barriers between the different disciplines and foster interdisciplinary communication and collaboration (8–10).

Educational pathways, such as MD/PhD-programs, have been developed to encourage training of physician-scientists. However, obtaining both a clinical and research degree does not necessarily create a physician-scientist, as clinical and research degrees are based on approaches that are fundamentally different (11). Many dual-degree programs lack integration of these different ways of thinking. Often, the full breadth of translational medicine, the perspectives of academia, industry, government, and patients, and the skills needed to work in multidisciplinary teams are not addressed. In addition to dual-degree programs there is a wealth of postgraduate training programs that focus their content on knowledge of translational medicine. The majority of these programs do not address the full range of competencies needed to become a lead figure in translational medicine. They often focus on a more specific skills set, such as postgraduate research training for clinicians or programs that focus on biomedical technology (10, 12, 13).

Most programs lack role modeling and mentorship (11, 14). Although mentorship for medical students is generally deemed important, students aiming for a translational scientist career are particularly in need of role models and mentors, because of the many challenges they need to face during their careers (15). It is often difficult to find a clinical job that allows for protected time for research, and funding and reward systems focus on publications and citation scores while translational research consists of longer periods that produce no or only lower-impact papers (16). Because of these challenges in the training and career pathways for translational scientists, the number of researchers and clinicians pursuing such a career has been static over the past decades, with an average age that continues to rise. All in all, regardless of the need for a growing number of translational scientists to advance medicine, this professional figure seems to be in danger of becoming extinct (16, 17).

The Eureka institute for Translational Medicine (http://www.eurekainstitute.org/) was founded in 2007 based on the realization that international, system-wide networks to train and sustain translational scientists did not exist. The Eureka institute aims to build an interdisciplinary community of translational professionals that are equipped to promote the development of true translational studies. The international certificate course that is organized on a yearly basis addresses the educational needs of Eureka's mission. During the course, participants are educated in knowledge domains of translational medicine through formal (e.g., workshops and seminars) and informal (networking opportunities with faculty) curriculum elements, and with mentoring from experts in translational medicine and education. Course evaluations directly after the course revealed high scores for both formal and informal curriculum elements. Participants reported a “paradigm shift” in their scientific knowledge and beliefs. The present study aims to investigate whether alumni of the Eureka certificate course are indeed better able to engage in translational research activities and how this can be supported. The research questions are: (1) *Are alumni of the Eureka certificate course better able to engage in translational activities?*, (2) *What conditions for change hamper or support Eureka alumni's engagement in translational research activities?* and (3) *What are the learning outcomes for Eureka alumni that impacted (one of) their professional activities (e.g., research, clinical work, education, management)?*

## Theoretical framework

The learning outcomes of the Eureka certificate course were expected to be complex in nature because the course curriculum covers various types of knowledge, for instance explicit knowledge regarding the health professions and personal knowledge of (partly) tacit nature. Further, the course builds forward on prior training and work experiences of the participants and combines knowledge and skills development. Therefore, personal professional theory (PPT) was used to understand the learning outcomes for Eureka alumni. The PPT concept was investigated by Schaap et al. (18) in the field of competence-based vocational education, where moving toward a competence-based model brought along issues with the definition and assessment of learning outcomes in which knowledge, skills, and attitudes were addressed as integrated wholes (19). Schaap et al. (18) define a PPT as “a personal professional knowledge base that serves as frame of reference in the process of internalizing professional knowledge and beliefs.” This process of internalization requires critical reflection on previous experiences, knowledge, and beliefs, and adopting shared knowledge and collective norms, values and beliefs of a vocational community so that they become personalized. The content of PPTs involves propositional knowledge, conceptual knowledge and personal beliefs. Propositional knowledge consists of discipline-based theories and concepts (20). Conceptual knowledge contains knowledge about facts, concepts and principles that can be applied in a specific domain. Personal knowledge is what an individual knows and is able to do (20). PPTs can act as a frame of reference through which new knowledge and beliefs can be acquired and interpreted and direct professional behavior.

The content of a PPT is divided into six objects: vocational domain, organizations, social environment, target group, technical-instrumental processes, and professional development. Together, these objects encompass the vocational knowledge, including knowledge on the professional environment and professional development, that is needed to perform adequately in a specific vocation (18).

## Methods

### Context

The Eureka institute defines translational medicine as the continuum from a scientific idea or finding to a diagnostic tool and/or therapy applied to human diseases. This means that translational scientists need to have a comprehensive understanding of the aspects of the translational process, including molecular medicine, intellectual property, financing, regulation, and pre-clinical and clinical studies, without necessarily being a specialist in any of those fields. Knowledge and beliefs of these different domains and disciplines are acquired during the certificate course through seminars, workshops, and case-studies that are facilitated by leaders in translational medicine and educational experts. As it remains impossible for one single person to be an expert of all aspects of the translational itinerary, the course also largely focuses on developing the skills to navigate this itinerary, namely communication, networking, and connecting the different domains and disciplines. Teambuilding activities and group assignments are designed to develop the skills to foster innovative teams, critical thinking, problem solving, and communicating effectively across broad audiences. Furthermore, the Eureka course has a personalized learning approach in which challenges and experiences of the participants are central in all sessions. Mentoring and speed-dating sessions with faculty are focused to provide individual advice on issues the participant raises from their working environment or related to career development.

The Eureka certificate course was first organized in 2009 and aims at mid-level career professionals who are working in the field of translational medicine. It is organized on an annual basis for an international group of around 30 participants and lasts 1 week. For the present study, all alumni who attended the certificate course from 2009 up to and including 2014 were approached (144 alumni in total).

### Design

An explanatory, mixed-methods design was selected and conducted in two phases (21). In the first phase a questionnaire was distributed among all 144 alumni of the Eureka certificate course. A questionnaire was chosen as the preferred method in the first phase because it would provide quantitative data on the number of alumni that indicated that their participation in the course had helped them to engage in translational activities. The responses to this questionnaire informed the development of a semi-structured interview guide and coding schemes for the second phase of this study. The interview approach was chosen to gain a better understanding of the conditions for change that hampered or supported the engagement in translational research activities and the learning outcomes for Eureka alumni that impacted their professional activities. Ethical approval for this study was obtained from the Ethical Review Board of the Netherlands Association for Medical Education (NERB#403).

### Participants

From 2009 to 2014, 144 participants took part in the Eureka certificate course. In March 2015 the questionnaire of the first phase of this study was sent to all 144 alumni. Seventy-eight alumni (54%) completed the questionnaire. In October 2016 the same 144 alumni were invited over email to participate in a semi-structured interview. In total, 14 alumni (8 male, 6 female) volunteered to participate, representing a variety of institutions from four continents. They were working as either a physician-scientist, full-time scientist, or manager of (translational) research. Fictitious names are used for quotes throughout this paper to indicate the gender of the alumni.

### Instrumentation

The questionnaire was developed by three of the researchers (BP, NR, and MW). Two of them (BP and NR) are experts in the field of translational medicine and one (MW) is the primary researcher and is not related to the Eureka institute. The questionnaire was pilot tested with five alumni of the Eureka certificate course. The input from these alumni led to minor changes to the wording of the questions. The final questionnaire consisted of the questions: *Were you able to apply what you learned at the Eureka course in your home environment? If yes, what allowed you to? If no, what prevented it?* and *Did your experience at the Eureka course change the jobs you've held or what you're doing in your work? If yes, please explain what changed?*

The semi-structured interviews were developed by the same three researchers and informed by the results of the questionnaire. The interviews covered 12 questions regarding learning outcomes of the Eureka course, intentions for practice and changes in practice after the Eureka course, conditions for change to engage in translational activities, and questions related to the interviewees engagement in translational networks.

### Data collection

The final questionnaire was distributed via email to alumni who had participated in the course anywhere from 1 to 6 years (mean 2.9 years, SD 1.6 years) since completion. The questionnaire was anonymous and all respondents provided informed consent before starting the questionnaire. The interviews were conducted by one of the researchers (MW) who is not associated with the Eureka institute, varying from two to seven years after alumni's participation in the course. Eleven of the 14 interviews were completed using Skype technology and three were conducted face-to-face. All interviews lasted up to 1 h, were audio recorded with the permission of the interviewees and transcribed verbatim without identifying data. The final questionnaire, interview scheme and coding schemes can be found in the Appendix.

### Data analysis

The transcripts of the semi-structured interviews were coded and analyzed by one of the researchers (MW) together with a research assistant. Both were not associated with the Eureka institute. Two coding schemes were developed, using directed content analysis methodology (22). This means that coding schemes were developed before the start of data analysis using prior research (coding scheme I) or existing theory (coding scheme II) (22). Coding scheme I regarded the second research question and thus focused on conditions for change that underlie the engagement of Eureka alumni in translational research activities. For the development of this coding scheme the descriptive qualitative responses to the questionnaire were coded, which led to the identification of six conditions for change. Coding scheme II concerned the third research question. This scheme was developed based on the work of Bakkenes et al. (23) in which four main categories of learning outcomes for teacher learning were defined and validated (as opposed to student learning in which case learning outcomes are often conceptualized as exam or test scores). The four categories of learning outcomes in our coding scheme II were: changes in knowledge and beliefs, intentions for practice, changes in practice, and changes in emotions. Each learning outcome category was subdivided into the six objects that form the content of a PPT: vocational domain, organizations, social environment, target group, technical-instrumental processes, and professional development (18). The initial coding schemes were applied by both coders independently to four randomly selected interviews (28%). Segmentation was initiated at utterance level (24). After each interview both coders met to compare the results of their coding, resolve differences by consensus discussion, and further develop the coding schemes. Coding scheme II only required minor changes for clarification in the operationalization of the codes. Coding scheme I was further expanded due to new themes that emerged, leading to the definition of additional codes, namely “(lack of) personal characteristics,” “(lack of) training in how to engage in translational research activities,” “(lack of) supportive funding and reward system,” and “(lack of) feasibility to conduct translational research.” After coding of the first five interviews was completed no new codes emerged.

The final coding schemes were checked for reliability in coding by determining interrater agreement. Both researchers independently coded two more interviews to account for more than 10% of the data (25). Interrater reliabilities were calculated separately for each coding scheme and showed an adequate level of agreement (inter-rater reliability 71 and 81%, and Cohen's kappa 0.77 and 0.88 for coding schemes I and II, respectively) (26). The final coding schemes were applied to the remaining seven transcripts by the research assistant.

## Results

### Questionnaire

Eighty-six percent of the alumni indicated that they had been able to apply what they learned at the Eureka certificate course in their (professional) home environment. For 51% participating in the Eureka course had played a role in the decision to change to a different job or in the way they were accomplishing their everyday work.

### Interviews

#### Conditions for change

One aim of the interviews that were held with 14 alumni was to understand the conditions that made it either possible or impossible for Eureka alumni to engage in translational research activities. This led to the identification of ten conditions for change underlying their engagement in translational activities, which are summarized in Table 1 and will be described in more detail below. All conditions for change start with “(lack of)” to indicate that the presence of the conditions supports engagement in translational activities while the absence of a condition hampers thisengagement. For most conditions, both absence and presence of a condition had been experienced by different alumni.

**Table 1:** Conditions for change underlying the engagement in translational research activities of alumni of the Eureka certificate course.

1.	(Lack of) latitude to conduct translational research
2.	(Lack of) motivation to conduct translational research
3.	(Lack of) opportunities to network and/or collaborate
4.	(Lack of) research time and/or money
5.	(Lack of) supportive professional partners
6.	(Lack of) translational work environment (general)
7.	(Lack of) personal characteristics
8.	(Lack of) training in how to engage in translational research activities
9.	(Lack of) supportive funding and reward system
10.	(Lack of) feasibility to conduct translational research

##### (Lack of) latitude to conduct translational research

Alumni described how having the opportunity to initiate research projects and collaborations was an important determinant for their ability to engage in translational research. Others, on the contrary, pointed out how they felt restricted to do so within their professional environment.

“I think my job is quite, I mean, I'm still within a 5 year contract that was very much, let's say, set in stone and it was clear what I was supposed to achieve and what to do. And I don't think I had any power to change this.” (Maria)

##### (Lack of) motivation to conduct translational research

The wish to contribute to better patient outcomes was described as a great motivator for almost all of the alumni. Doing research with the patient in mind was said to give additional meaning and relevance to their work and felt more rewarding than research without clinical application.

“I still want to fight for a better outcome for my patients so I keep on doing this, and I like it, you know, if you don't like it you're not going to stay in this game.” (Anna)

##### (Lack of) opportunities to network and/or collaborate

The importance of being able to contact other people in the field of translational medicine was emphasized by most of the alumni. Examples of experienced benefits were finding a new job through contacts within the alumni's network, knowing more senior translational professionals who can act as mentors, receiving input on research proposals from peers, establishing collaborations for research and educational activities, and being stimulated and inspired by people who share the same objectives.

The lack of a collaborative atmosphere, or more specifically a competitive atmosphere, was said to be counterproductive as it leads to delays in research and increases in research costs.

“So that is someone who has more power for his experiments, but we believe that over there they draw conclusions too quickly. But if we could collaborate, we could talk about these things and exert some influence. And then we would not have to spend money on the same research twice.” (Julie)

##### (Lack of) research time and/or money

Having protected time for research was regarded as an essential factor by most of the alumni. Although this seems to be most obvious for clinician-scientists, as clinical duties often take priority over research, also fulltime scientists experience a lack of time due to teaching and management obligations. Job profiles that prescribe a certain percentage of time that is (contractually) protected for research seem to be successful examples in some institutions. The issue of funding for research was often related to the issue of time, as being dependent on grants for research funding is time consuming, especially with success rates that have gone down considerably over the years. Working in labs that have sufficient funding and institutions that provide support for early-career researchers or start-up funding were therefore considered to be very helpful.

“So there's start-up funding that was made available to me both from the hospital department of pediatrics and from the research institute, and that has been critical, because as I said grant funding is hard to get and when it runs out it runs out and then there is nothing. Yet you have a lot of fixed costs…yeah.” (Luke)

##### (Lack of) supportive professional partners

Alumni described how the support of professional partners was very important for their ability to engage in translational research. Partners that were mentioned were superiors, clinicians, researchers, colleagues from different departments or disciplines, mentors, and students.

“I have my position, my current position now in large part because I entered a purely clinical division and they were very interested in having a research component, like a science translational component […] and so that team, you know, was very open to having me join them and they have been very strong advocates for me and I wouldn't have this position without them.” (Laura)

##### (Lack of) translational work environment

Many examples were given during the interviews of how a “translational philosophy,” and the presence of “visionary people” who contribute to the realization of such a translational work environment, influence the alumni's ability to engage in translational research. One such example is having established collaborations between hospitals and universities as it provides an infrastructure that enables translational research: it is easier for clinicians and scientists to collaborate and understand each other, improves access to data and (clinical) samples, and provides more opportunities for patients to participate in trials from which they may benefit. Furthermore, it prevents clinician-scientists from feeling torn when they have to choose whether an article or grant should count for the hospital or the university.

Supportive Human Resource Management practices and support for early career researchers were also mentioned, as is exemplified in the following fragment:

“There are moments in our career when we need more support and that will pay of later on. But…at an early stage of your career, that you have to be as good as a well-established professor in terms of, you know, bringing revenues for research and publishing, that's a bit unfair.” (Maria)

##### (Lack of) personal characteristics

Most alumni mentioned a number of personal characteristics that are required to succeed in translational research. They mentioned that translational professionals need to be very good communicators and collaborators that function well in multidisciplinary teams, rather than striving to succeed in individual, goal driven research. Since many described how working in translational medicine can be challenging and stressful, commitment, perseverance, time-management and being able to ensure a good work-life balance were also mentioned as important factors for success.

“I see these as people who have a cohesive team around them. They have a really clear focus on an important health issue. They know how to communicate well, so for example, there is a group who is getting huge amounts of funding for diabetes, you know translational work in diabetes, huge amounts, millions of dollars, but they have really, they've got their collaborators from all over the country, they're actually bringing all the people in that they need, they've got a really tight team around them, they've brought in all the assets that they need to make their work happen. So they're quite entrepreneurial in their approach. The people who are less entrepreneurial, so who are much more inward looking, are not as successful.” (Emma)

##### (Lack of) training in how to engage in translational research activities

The issue of training in translational research was mentioned in several interviews. Some said that it was not until their participation in the Eureka certificate course that they gained a full understanding of the translational pathway. Looking back, they would have liked to have received this kind of training earlier on in their careers, for example during graduate training or while working on their PhD. Others noted how difficult it is to find students who are interested in translational medicine because they are very “polarized” when they finish their undergraduate degrees due to the focus of these programs on either basic science or medicine. For students who do pursue a combined training path, such as an MD-PhD program, alumni felt it was difficult to see the goal at the end of that pathway due to limited opportunities to work as clinician-investigator or clinician-scientist.

“But I also think that training is really important, and I think potentially even introducing new approaches to PhD training, so really starting to train young researchers earlier and not just young researchers, but young clinicians, you know, really bringing them together with researchers, to work out how do they prioritize the questions that they are asking, and how do they achieve the best outcome for their patients? Because that's what they want, that's what the researchers and clinicians want, that's the thing that drives them.” (Emma)

##### (Lack of) supportive funding and reward system

Current funding and reward systems that focus on prominent author positions on high-impact papers were often seen as a difficulty in succeeding in translational research. As successful translational work is often the result of a collaboration between scientists and clinicians, and often additional partners, metrics in terms of author positions on papers and impact factor do not adequately reflect the work that was put in. One interviewee called the current system “anti-collaborative” and “anti-translational.” Many alumni felt that every author position on a paper should be valued and that, in addition to publications, translational outcomes should be demonstrated and rewarded.

Some alumni also described how they felt restricted to engage in translational activities by these systems as grants and job evaluations often depend on these author positions, number of publications and impact factor. Because that is the case, they felt pressed to spend time on projects that are less translational, but lead to faster results and can be published in higher impact papers.

“Because at the end of the day, no matter if someone is in industry or somebody is in university, we all answer to somebody and if we don't answer to that person or entity the way that we need to, we cease to have the position. You know, so there's always conflicting priorities. So I think it would be brilliant if there could be some shift away from, in my world academics, the traditional metrics of publications, grants, and presentations to something that values collaborative work and ideas more than it is today. And now I feel that it's only valued when it turns in to the traditional types of academic output, which inherently puts a constraint on even the kinds of translational ideas that you can think about.” (Luke)

##### (Lack of) feasibility to conduct translational research

Some alumni mentioned factors that were difficult to influence, but could determine whether their efforts would be successful. Examples include ending up at the right institution, meeting the right people at a conference, and ending up with a patentable discovery.

“So I was really at a moment in my life where I was questioning where I wanted to go and the truth is, a friend saw the advertisement for [my current position] and said it is not for me but maybe you should look at it, because it might be interesting for you. And I looked at it and I liked what they were doing and the position, so I applied but it was really by chance. Also, I think at that time I was ready for a next move in my career, it came at a point where I was ready to take this step.” (Sophie)

#### Learning outcomes

In this part we describe how the learning outcomes of the alumni of the Eureka certificate course contributed to their engagement in translational activities, which was the second aim of the semi-structured interviews. Results are described for each of the four categories of learning outcomes, and – when applicable – each of the six objects of PPT.

##### Changes in knowledge and beliefs

*Vocational domain* Almost all alumni reported that obtaining a clear perspective on the full spectrum of translational medicine was one of the most valuable outcomes of the Eureka course. Alumni were generally not aware of the entire translational process prior to the course or had used the term “translational research” for different types of research, as one alumnus explains:

“It really struck me that that's a mistake I think is often made, and this phrase ‘translational research' is really misinterpreted and misused very frequently. So I used to say that I did [do translational research], but actually what I was doing was basic research that was with some human cells now and then, rather than kind of thinking through, you know, a much more complex process, which is actually what translation is.” (Tom)

This insight in the full translational pathway influenced what alumni regarded as end point of their research and how they felt about the need for inter-professional collaborations. Gaining a better understanding of the drug development pathway, the importance of intellectual property (IP) and patenting in order to be an interesting partner for industry, and the importance of interfacing with people who are making (health) policy decisions directed their focus toward the implementation of research outcomes, rather than publication of results only.

“So I think where Eureka has influenced my thinking maybe is what I'm going to do with the results of my studies and how I'm going to think about translating that into, you know, something beyond just ‘here's the paper reporting the results'.” (Luke)

Moreover, the clear perspective on the full translational spectrum and stakeholders involved allowed participants to reflect on their own role and where they wanted to be within that spectrum:

“I think since Eureka, you know I'm very clear that I'm a, you know I'm a basic and a translational scientist, that I started to use that word and feel more comfortable saying that you know, that I‘m a translational scientist, that translation isn't just something you do. You can actually sort of be that person that takes care of that type of research.” (Laura)

*Organizations* One alumnus said that the personalized approach of the Eureka course led to new insights in how to manage people in research:

“For me, the most important insight was actually the way the course was set up, connecting the human dimension, like personal growth and development and how people interact with each other as persons instead of professionals, to connect that to the challenges in the field research.” (Alex)

*Social environment* For most alumni the course helped to gain an overview of the people involved in translational medicine, which helped to understand the need to collaborate with people and with organizations.

“I have a much more well thought out understanding of how this all works and where I fit within it, and what needs to be done for me to make a connection, if I need to make the connection, and how to build bridges. So I think you know Eureka provided a lot of time for thinking and time for talking to people from different areas, who have, even though they are form different areas, have similar experiences and similar frustrations and road blocks, and I think it gained a lot more insight.” (Luke)

Furthermore, the interaction with other participants fostered a greater understanding of translational professionals from different backgrounds.

“I think that, to understand what motivates different people, you know what's motivating a scientist vs. a clinician when they approach a problem, understanding how those sort of, our training makes us sometimes good collaborators, and not so great collaborators.” (Laura)

*Personal development* Alumni described that the course showed them the difficulties in communication and collaboration, which enabled them to reflect on their own communication and collaboration styles.

“It provided me a lot of insight in the way people can behave very different in a group […] and how that's fine. So it is important to have diversity within a group and for everyone to have different characteristics.” (Julie)

##### Intentions for practice

*Vocational domain* A few alumni described how, immediately after the course, they had planned to look back at projects they had undertaken in the past to see whether the results could be translated into clinical practice. In general, however, alumni seemed to have little recollection of the intentions they had at the end of the course. Other intentions had turned into changes in practice by the time of the interview and will be discussed under 1.3.

*Social environment* The network of alumni of the Eureka certificate course was mentioned during all of the interviews. Almost all alumni mentioned that they wanted to stay in touch with the Eureka community, usually because of a combination of the friendships that had formed during the course and the want to be in contact with peers or mentors. Although most alumni were still in contact with at least some of the Eureka alumni or faculty, it was also indicated that they did not form a cohesive network. Possible explanations that were provided during the interviews were physical distance (as the Eureka alumni form an international group), restrictions in time, limited follow-up by the Eureka institute, and the difficulty of connecting over a common theme that is as broad as translational research without a specific project binding them:

“And at least in my case, whenever I've built meaningful professional connections, that have blossomed into something long-term, and actually had tangible benefits, it's always been or almost always been around specific work that we've done together. As opposed to just ‘hey you're an interesting person in a different discipline, let's translate together'.” (Kevin)

##### Changes in practice

*Vocational domain* Three different types of changes in practice within the vocational domain were described by the alumni: changes in professional appointment, changes in research activities, and changes in teaching styles or methods.

Participating in the Eureka course led to a change in professional appointment for some of the alumni, because they felt restricted in their abilities to do translational research in their previous positions. These changes in positions were either within academia toward a more translational environment, or from academia to industry as is the case in the following example:

“So I've actually very recently accepted a new position at a company, and that has certainly been influenced by my experience at Eureka. And so from January I'll be moving to a technology development company that is more focused on translation, you know from a bit more commercial rather than an academic side, but it's what I want to do because I am so interested in actually delivering something that you know success or fail, at least you take it to those steps to test that. And that definitely has been Eureka, has had an impact on that decision.” (Tom)

Others described how their research activities have become more translational, for example by setting up collaborations with clinical departments to be able to use clinical samples rather than animal models or by deliberately choosing research projects that may benefit patients over research projects that may lead to publications on the short term.

Alumni also reported changes in practice outside the translational domain: specific teaching methods and elements of the personalized teaching style that is applied during the Eureka course have been used by alumni in their own teaching activities and in interactions with colleagues and other people. Also, some alumni organized courses and workshops that were inspired by the Eureka certificate course.

*Organizations* Alumni described how the Eureka course helped them to create a research team, acknowledging what people did for the team and helping them to develop themselves. One alumnus described how he restructured a research department:

“We work with approximately 40 researchers in the lab and 40 clinical researchers, and then the clinical department is even bigger. So I think the setting up a structure in which people, despite them working on very different topics without speaking one another's language, do collaborate and believe it to be an integrated and meaningful experience, that is something that I, for the better part, gained from the Eureka course.” (Alex)

*Personal development* Alumni described how they gained the skills to communicate around the impact and relevance of their work, to reflect on their careers and take leadership in professional decisions, thanks to the confidence they gained in their roles as translational professional during the Eureka course.

##### Changes in emotions

*Vocational domain* Alumni described that the course increased their motivation to become translational professionals, because they felt inspired by the faculty and other participants, who showed them that it was possible to succeed in translational medicine, and because of the inspiration for new projects and possibilities to make their own work more translational. For others, the Eureka course came at a time where they were deciding on future directions for their careers, for which the Eureka course offered them the ideas, contacts or confidence.

## Discussion

This study set out to investigate whether alumni of the Eureka course were better able to engage in translational activities, the conditions for change that hampered or supported Eureka alumni's engagement in translational research activities, and the learning outcomes of the Eureka certificate course that impacted their professional activities.

Two to seven years after the course alumni reported high impact of the course on their professional activities, both in terms of applying what was learned (89%) as well as on job crafting (51%). Though by no means of proof of the efficacy of the course, this finding is remarkable in the light of what we know about the professional struggles of translational scientists.

Ten conditions for change were identified that had either hampered or supported the engagement of alumni in translational activities. Two conditions that mainly hampered this engagement focused on the lack of (dedicated) time and funding for research and the current funding and rewards systems. These issues have frequently been addressed in the literature (3, 16, 27). Research requires protected time, but often this time is limited due to patient care, management activities or teaching expectations. Funding for research largely depends on grants, which further draws away from the already limited time for research. Further, academic promotions are ultimately based on publications, citation indices, and related metrics such as the Hirsch-index, discouraging publishing on the implementation of research findings in practice as this type of research takes considerable more time to produce (3, 16). The results of the questionnaire, however, indicated that a large number of alumni succeeded to engage in translational research despite these systems. Our results suggest that this was likely due to a combination of conditions that are supportive for engaging in translational activities, such as supportive professional partners and working in a translational work environment, and the learning outcomes that resulted from alumni's participation in the Eureka certificate course.

The concept of PPT was used to understand the learning outcomes and how they enabled alumni to further develop as translational professionals. Gaining a full understanding of the whole translational pathway and the stakeholders that are involved in this pathway seem to be the most important insights that alumni gained from the Eureka certificate course. It enabled them to reflect on their own role within that pathway and stimulated them to more consciously make decisions on the type of research they wanted to engage in, the environment they wanted to work in, and the people they wanted to collaborate with. Moreover, it was mentioned how the course gave them the skills and confidence to pursue a career as a translational professional. Alumni indicated how they would have liked to gain this insight earlier on in their careers and addressed the need for more education on translational research in graduate training and PhD programs.

No learning outcomes were reported in the objects “technical-instrumental processes” and “target group.” This is likely due to the fact that this type of knowledge falls outside the scope of the Eureka course, but rather is addressed during prior (bio)medical training or PhD tracks. These objects focus on discipline specific knowledge, while the participants of the Eureka course represent a diverse and multidisciplinary group.

A remarkable outcome was the number of alumni who mentioned how the teaching style of the Eureka course had impacted their own teaching activities. For some alumni it had influenced the way they were interacting with colleagues or organized a research unit.

This study was not set up to compare the Eureka certificate course with other graduate and postgraduate training programs on translational research. Still, a number of differences can be observed. Many of the shorter courses focus on technical skill development, business management and leadership, or knowledge on the translational spectrum without integrating this knowledge with interdisciplinary skills development, mentoring, and community building. For most of these programs data regarding the long-term impact of these courses for comparison are not (yet) available (10, 12, 13). Other programs that do combine multiple components are often master or postgraduate training programs of much longer duration, varying in length from multiple weeks up to 6 years. An example is the National Institutes of Health (NIH) Clinical and Translational Science Award (CTSA) mentored career development program (https://ncats.nih.gov/training-education) that supports translational scientists in the transition from mentored to independent research funding (28).

Building a community of interdisciplinary translational professionals, one of the goals of the Eureka institute, seems to be the most challenging. Although most alumni were still in contact with at least some alumni or faculty this has not led to the formation of a structured network yet. As fostering community to prevent isolation has often been described as a necessity for translational scientists (29–31) this is an aspect that can still be improved and may contribute to a further increase in alumni's ability to engage in translational activities.

This study has a number of limitations. Although the use of a questionnaire and interviews was deemed most suitable to address our research questions, this may have led to a response bias in favor of alumni who benefitted most from their participation in the Eureka certificate course. Also, our questionnaire and interviews focused on the perspectives of the alumni. The outcomes of the course were not observed or measured. Due to the explanatory nature of this study we do however feel that this did not have substantial impact on our results.

## Conclusion and recommendations

Current translational medicine needs translational professionals with a broad set of knowledge and skills to help breaking down the barriers between the different disciplines that are involved in the translational pathway. Becoming such a translational professional, however, is challenging due to the lack of training programs, the current funding and reward systems, and the lack of support for (especially early-career) translational professionals in terms of start-up funding and dedicated time for research. Although these systems are influenced by economical, political, social and cultural factors (3) and are therefore not easily changed, this study showed that education in translational medicine can have a large impact on the careers of translational professionals. Together with the conditions for change that have been identified in this study this may enable young translational professionals to succeed in their translational activities, and thus help to close the gap between biomedical research and its clinical application, and reduce the waste in research funding.

## Author contributions

MW, BP, and NR designed the study and developed the questionnaire and semi-structured interview guide. MW analyzed the results together with a research assistant. MW wrote the first draft of the paper, with extensive feedback from MvdS and BP. All authors provided critical comments and revisions and approved the final manuscript.

### Conflict of interest statement

MW, MvdS and MK declare no conflict of interest. BP, NR and JH are involved with the Eureka Institute for Translational Medicine and have therefore not participated in either data collection or data analysis.
