# Supplementary material for: Preventing Translational Scientists From Extinction: The Long-Term Impact of a Personalized Training Program in Translational Medicine on the Careers of Translational Scientists
Source: Front Med (Lausanne). 2018 Nov 9;5:298. doi: 10.3389/fmed.2018.00298 (PMC6237913; doi:10.3389/fmed.2018.00298)
Supplement: Supplementary file 1 [file Data_Sheet_1.docx]

**Appendix I. Questionnaire and semi-structured interview guide**

**Questionnaire**

1. Were you able to apply what you learned at the Eureka course in your home environment? If yes, what allowed you to? If no, what prevented it?
2. Did your experience at the Eureka course change the jobs you've held or what you're doing in your work? If yes, please explain what changed?.

**Semi-structured interview guide**

1. What is your personal evaluation of the Eureka certificate course?
2. At the end of the Eureka course, did you plan to change anything?
3. Did Eureka change the way you look at things in your everyday work?
4. Are there things you are doing differently now then at the time of attending the Eureka course?
5. Were there things you wanted to do or change but could not?
6. Do you feel part of a translational network?
7. Do you see yourself as a translational scientist / clinician-scientist?
8. In general, what do you think it takes to become a translational scientist / clinician-scientist?
9. What helped you to get where you are now?
10. Are there also things that blocked you from what you planned or wanted to do?
11. Is there anything else you would like to say that wasn’t asked?
12. Do you have any other suggestions for the Eureka course?

**Appendix II. Coding schemes**

**Coding scheme I – Conditions for change**

| **Code** | | **Operationalization** |
| --- | --- | --- |
| The codes in this codebook refer to conditions identified by the interviewee, that make it (im)possible for them to engage in translational research activities, which may or may not be an outcome of the Eureka course. The identification of these conditions need to be based on their own experiences (not on those of others). | | |
| 1. | (Lack of) latitude to conduct translational research | This code is met if a segment refers to  (a) opportunities to engage in, or the autonomy to initiate, translational research activities within the professional environment of the interviewee,  (b) a lack of opportunities to engage in, or lack of autonomy to initiate, translational research activities within the professional environment of the interviewee. |
| 2. | (Lack of) motivation to conduct translational research | This code is met if a segment refers to  (a) an interviewee’s motivation to engage in translational research activities within his/her professional environment,  (b) an interviewee’s lack of motivation to engage in translational research activities within his/her professional environment. |
| 3. | (Lack of) opportunities to network and/or collaborate | This code is met if a segment refers to  (a) opportunities to network and/or collaborate on a local, national, or international level with (a) other Eureka alumni, (b) other professionals in the field of translational medicine, or (c) important (social) actors in the work process (e.g., patient organizations, industry, governmental institutions, non-profit organizations),  (b) a lack of opportunities to network and/or collaborate on a local, national, or international level with (a) other Eureka alumni, (b) other professionals in the field of translational medicine, or (c) important (social) actors in the work process (e.g., patient organizations, industry, governmental institutions, non-profit organizations)  (c) the presence of a collaborative atmosphere (both within and between institutions) and/or a cohesive team within the institution (i.e., “happy lab”)  (d) the absence of a collaborative atmosphere (i.e., a competitive atmosphere; both within and between institutions ) and/or a cohesive team within the institution |
| 4. | (Lack of) research time and/or money | This code is met if a segment refers to  (a) sufficient or protected time and/or sufficient funding to engage in translational research activities ,  (b) insufficient or unprotected time and/or insufficient funding to engage in translational research activities. |
| 5. | (Lack of) supportive professional partners | This code is met if a segment refers to  (a) the presence of one or more professional partners (e.g., superiors, peer scientists, clinicians) within the professional environment who support the interviewee in engaging in translational research activities,  (b) the absence of one or more professional partners (e.g., superiors, peer scientists, clinicians) within the professional environment who support the interviewee in engaging in translational research activities.  N.B. The most important difference between codes 3.5 and 3.6 is then that code 3.5 refers to direct (non-)support from *people*, whereas code 3.6 refers to (non-)support stemming from the *infrastructure* that is in place within the interviewee’s professional environment. |
| 6. | (Lack of) translational work environment (general) | This code is met if a segment refers to  (a) a professional environment that supports the interviewee to engage in translational research activities. This environment has to be there or the institution one works in has to be sufficiently engaged with translational research to want to create all of it (c.f. Kevin p.7-8).  (Examples of such an environment could be: a translational “philosophy” within the professional environment, a shared vision between hospitals and universities (and possible other stakeholders), access to data across hospitals and universities, access to high quality staff (e.g. to conduct experiments), supportive HR(D) practices, and visionary “drivers” who contribute to the realization of such a translational work environment),  (b) a professional environment that does not support the interviewee to engage in translational research activities. This environment is not there or the institution one works in is not sufficiently engaged with translational research to want to create all of it (c.f. Kevin p.7-8).  (Examples of the lack of such an environment could be: the lack of a translational “philosophy” within the professional environment, no shared vision between hospitals and universities (and possible other stakeholders), no access to data across hospitals and universities, no access to high quality staff (e.g. to conduct experiments), the lack of supportive HR(D) practices, and the lack of visionary “drivers” who contribute to the realization of such a translational work environment).    (HR = human resources; HRD = human resource development) |
| 7. | Other | This code is met if a segment cannot be coded using codes 3.1 up to and including 3.6, and codes 3.8 up to and including 3.11. In such a case, we assign code 3.7 and add a memo in which we propose a new subcode. |
| 8. | (Lack of) personal characteristics | This code is met if a segment refers to  (a) the presence of certain personal characteristics (who you are and what you are capable of, e.g. thinking logically, asking the right questions) that enables someone to conduct translational research,  (b) the absence of certain personal characteristics (who you are and what you are capable of, e.g. thinking logically, asking the right questions) that hinders conducting translational research  If this code is assigned we add a memo describing the specific personal characteristic (e.g. entrepreneurship, excellence, perseverance, social skills, collaborative skills). |
| 9. | (Lack of) training in how to engage in translational research activities | This code is met if a segment refers to  (a) educational activities that have contributed to one’s knowledge or understanding of translational research. These educational activities may have taken place before or after, but not during, the Eureka course,  (b) a lack of educational activities related to translational research before or after, but not during, the Eureka course. |
| 10. | (Lack of) supportive funding and reward system | This code is met if a segment refers to  (a) (monetary) recognition (e.g. grants) and/or other rewards (e.g. promotions) for (contributing to) translational research, for example in the form of research papers,  (b) the lack of (monetary) recognition (e.g. grants) and/or other rewards (e.g. promotions) for (contributing to) translational research, for example in the form of research papers. |
| 11. | (Lack of) feasibility to conduct translational research | This code is met if a segment refers to  (a) the presence of factors that enable translational research, such as clinically relevant research questions, patentable discoveries, and “luck”,  (b) the absence of factors that enable translational research, such as no clinically relevant research questions, no patentable discoveries (which might be due to earlier publishing instead of patenting discoveries), no “luck”. |

**Coding scheme II – Learning outcomes**

| **Code** | | **Operationalization** |
| --- | --- | --- |
| **1. Changes in knowledge and beliefs** | | This code is met if a segment refers to  (a) content that was valued as important and of which alumni were not, or less, aware of before attending the Eureka course (awareness),  (b) ideas or insights that already existed before attendance of the Eureka course, and that alumni felt more strongly about or which became more deeply rooted after the course (confirmed ideas), and/or  (c) ideas or insights that did not exist in this form before attendance of the Eureka course (new ideas). |
|  | a. Vocational domain | This code is met if a segment refers to  (a) relevant developments in a specific vocational domain (i.e., translational medicine),  (b) knowledge of laws and domain regulations,  (c) domain specific developments (e.g., neurology),  (d) general cultural values,  (e) vocational ethical codes, and/or  (f) knowledge of the environment of organizations. |
|  | b. Organizations | This code is met if a segment refers to  (a) the structure and/or  (b) the culture of organizations. |
|  | c. Social environment | This code is met if a segment refers to  (a) formal and informal relations,  (b) roles of colleagues or managers,  (c) power in environment organizations,  (d) important (social) actors in the work process (e.g., patient organizations, industry, governmental institutions, non-profit organizations), and/or  (e) social interaction with people in organizations.  N.B. This code includes segments referring to networking activities. |
|  | d. Target group | This code is met if a segment refers to knowledge that is directly used in interaction with the target group (i.e., patients), including knowledge of formalized proceedings to deal with complex problems of patients. |
|  | e. Technical-instrumental processes | This code is met if a segment refers to specific, technical, standardized vocational knowledge, found in specific instrumental procedures, standardized actions, or the use of specific instruments or tools. |
|  | f. Personal development | This code is met if a segment refers to personal development and learning (e.g., personal and professional skills not necessarily related to one’s own vocational domain such as presenting, collaborating, reflecting, and engaging in professional development activities), initiated in the Eureka course. |
| **2. Intentions for practice** | | This code is met if a segment refers to  (a) intentions to do things differently in the future (intention to try new practice),  (b) intentions to continue new practices with or without adjustments to improve them (intention to continue new practices), and/or  (c) intentions to continue current (old) practices. |
|  | a. Vocational domain | This code is met if a segment refers to  (a) relevant developments in a specific vocational domain (i.e., translational medicine),  (b) knowledge of laws and domain regulations,  (c) domain specific developments (e.g., neurology),  (d) general cultural values,  (e) vocational ethical codes, and/or  (f) knowledge of the environment of organizations. |
|  | b. Organizations | This code is met if a segment refers to  (a) the structure and/or  (b) the culture of organizations. |
|  | c. Social environment | This code is met if a segment refers to  (a) formal and informal relations,  (b) roles of colleagues or managers,  (c) power in environment organizations,  (d) important (social) actors in the work process (e.g., patient organizations, industry, governmental institutions, non-profit organizations), and/or  (e) social interaction with people in organizations.  N.B. This code includes segments referring to networking activities. |
|  | d. Target group | This code is met if a segment refers to knowledge that is directly used in interaction with the target group (i.e., patients), including knowledge of formalized proceedings to deal with complex problems of patients. |
|  | e. Technical-instrumental processes | This code is met if a segment refers to specific, technical, standardized vocational knowledge, found in specific instrumental procedures, standardized actions, or the use of specific instruments or tools. |
|  | f. Personal development | This code is met if a segment refers to personal development and learning (e.g., personal and professional skills not necessarily related to one’s own vocational domain such as presenting, collaborating, reflecting, and engaging in professional development activities), initiated in the Eureka course. |
| **3. Changes in practice** | | This code is met if a segment refers to  (a) behavioural changes (new practices), and/or  (b) behavioural changes in the opposite direction of what was taught at the Eureka course (back to old practices). |
|  | a. Vocational domain | This code is met if a segment refers to  (a) relevant developments in a specific vocational domain (i.e., translational medicine),  (b) knowledge of laws and domain regulations,  (c) domain specific developments (e.g., neurology),  (d) general cultural values,  (e) vocational ethical codes, and/or  (f) knowledge of the environment of organizations. |
|  | b. Organizations | This code is met if a segment refers to  (a) the structure and/or  (b) the culture of organizations. |
|  | c. Social environment | This code is met if a segment refers to  (a) formal and informal relations,  (b) roles of colleagues or managers,  (c) power in environment organizations,  (d) important (social) actors in the work process (e.g., patient organizations, industry, governmental institutions, non-profit organizations), and/or  (e) social interaction with people in organizations.  N.B. This code includes segments referring to networking activities. |
|  | d. Target group | This code is met if a segment refers to knowledge that is directly used in interaction with the target group (i.e., patients), including knowledge of formalized proceedings to deal with complex problems of patients. |
|  | e. Technical-instrumental processes | This code is met if a segment refers to specific, technical, standardized vocational knowledge, found in specific instrumental procedures, standardized actions, or the use of specific instruments or tools. |
|  | f. Personal development | This code is met if a segment refers to personal development and learning (e.g., personal and professional skills not necessarily related to one’s own vocational domain such as presenting, collaborating, reflecting, and engaging in professional development activities), initiated in the Eureka course. |
| **4. Changes in emotions** | | This code is met if a segment refers to  (a) positive emotions regarding translational research activities that were elicited by the Eureka course, such as feelings of pride, satisfaction, happiness, hope, courage, or positive expectation,  (b) negative emotions regarding translational research activities that were elicited by the Eureka course, such as feelings of irritation, anger, shock, fear, concern or doubt, and/or  (c) indications of unexpected revelation or disclosure (surprise). |
|  | a. Vocational domain | This code is met if a segment refers to  (a) relevant developments in a specific vocational domain (i.e., translational medicine),  (b) knowledge of laws and domain regulations,  (c) domain specific developments (e.g., neurology),  (d) general cultural values,  (e) vocational ethical codes, and/or  (f) knowledge of the environment of organizations. |
|  | b. Organizations | This code is met if a segment refers to  (a) the structure and/or  (b) the culture of organizations. |
|  | c. Social environment | This code is met if a segment refers to  (a) formal and informal relations,  (b) roles of colleagues or managers,  (c) power in environment organizations,  (d) important (social) actors in the work process (e.g., patient organizations, industry, governmental institutions, non-profit organizations), and/or  (e) social interaction with people in organizations.  N.B. This code includes segments referring to networking activities. |
|  | d. Target group | This code is met if a segment refers to knowledge that is directly used in interaction with the target group (i.e., patients), including knowledge of formalized proceedings to deal with complex problems of patients. |
|  | e. Technical-instrumental processes | This code is met if a segment refers to specific, technical, standardized vocational knowledge, found in specific instrumental procedures, standardized actions, or the use of specific instruments or tools. |
|  | f. Personal development | This code is met if a segment refers to personal development and learning (e.g., personal and professional skills not necessarily related to one’s own vocational domain such as presenting, collaborating, reflecting, and engaging in professional development activities), initiated in the Eureka course. |
